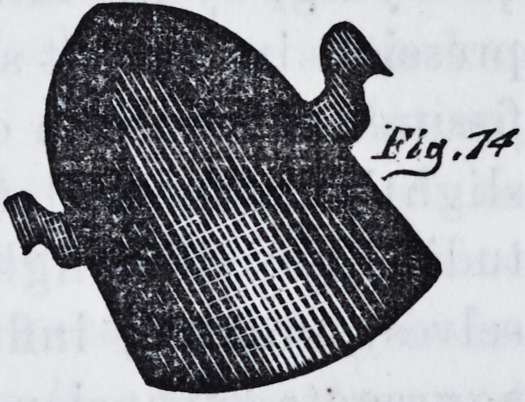# Improved Method of Casting Metallic Dies Direct from the Plaster Impression
*Owing to the length of Dr. House's interesting thesis, we have taken the liberty of publishing only those portions immediately relating to the new method.—Eds.


**Published:** 1860-04

**Authors:** J. Carroll House


### ARTICLE IV.

*Improved Method of Casting Metallic Dies Direct from the Plaster Impression.** By Dr. J. Carroll House.

Having thus dwelt, perhaps, at greater length than our present limits will warrant, upon some preliminary considerations of mechanical dentistry, we now proceed to discuss a method for procuring the metallic die, which shall, as nearly as possible, represent the organ of which it is the counterpart. And we may here be excused for remarking that the peculiar manipulations to be described originated with ourselves. (The casting of a die from the impression itself, as hereinafter mentioned, however, is not recent.) These we have constantly practiced for some

* Owing to the length of Dr. House's interesting thesis, we have taken the liberty of publishing only those portions immediately relating to the new method.—Eds.

years; and when we say that we have never been obliged to make over a single piece from any imperfection of adaptation in the base plate, we think that this statement of itself, will show the superiority of our method over any other now pursued by the profession. Many of the other methods have been tested in comparison with this one, and in every instance it has been proven the best. We refer now, of course, to simply swaged work.

We always take our impression of the jaw in plaster of paris, and in order to more complete success, we use the finest that can be procured. Having a good quality of plaster for taking the impression, it is necessary to use an impression cup, large enough for the impression to possess sufficient strength in every direction to permit of its detachment without endangering its safety. This may generally be done by reversing the cup, which may be made of Britannia metal, with the impression downwards, on a table, protected by a few thicknesses of cloth or paper, and rapping gently on the cup with some light instrument; by this means the cup becomes detached from the impression, isolated, and we are now in condition to proceed to the next step of our manipulations. As there is sometimes difficulty in procuring the kind of cup here referred to, those made of sheet tin may be recommended; but as the plaster, in hardening, attaches itself pertinaciously to the cup, the loosening of our impression becomes one of danger and difficulty. To coat the cup with a thin film of oil before placing the plaster in it, would indeed easily prevent this, but it gives rise to a greater evil, namely, the detachment of the cup in the act of removal from the mouth. This accident often happens in the use of any kind of cups. To obviate this last embarrassment we contrived a cup made according to the following directions:

Procure from the tinners some scraps of tin plate of different degrees of stiffness, and with a strong pair of plate shears, a small copper soldering tool, and some bits of solder, we have all the articles necessary. Proceed to cut a base plate of the form represented in fig. 1, and around the periphery of the arch, *a a*, turn up a shallow flange about the $\frac{1}{16}$ of an inch high, to act as a shoulder or support to the curve, and also to stiffen the plate. In this plate punch several holes, as at *b b*, about a quarter or three-eighths of an inch from the rim, and an eighth or $\frac{3}{16}$ of an inch in diameter; these holes are to be countersunk from above in such a manner as to allow the bevel to reach the under side. In the posterior part of the plate it will be observed that a portion of the plate is cut away. The space is covered by a raised cap (fig. 4) which is soldered around its edges to the base plate, thus giving the requisite prominence to the part which carries the plaster into the palatine arch. We now take a strip of thin plate, (fig. 2,) of such width as to give the proper depth to the cup, the edge of which, at *a a*, doubled upon itself, gives us a thickness at the upper edge precluding the possibility of lacerating the mucous membrane or facial mus-

Fig. 1

Fig. 4

Fig. 5

Fig. 2

cles. The strip is now bent, with the turned edge outward, so as to spring into and fit the flange around the base plate, and secured by solder. To give greater thickness and strength to the anterior projection, (*c*, fig. 1,) which serves as a handle for the introduction and withdrawal of the cup, we cut out a cap (fig. 5) and solder it around the edge, and also to the curve or rim; the piece being *raised*, as the tinners term it, by a round-faced hammer on a piece of lead, a slight convexity and consequently greater strength is given.

Beyond the holes in the plate we have only thus far given a description of the ordinary sheet tin impression cup, such an one as we might use in any upper case with wax or gutta percha, when it is desirable to carry the impression back to the posterior part of the jaw; but we have spoken of it thus in detail for the purpose of pointing out the improvements which give it a great advantage over the ordinary cup.

Having our cup properly constructed, we may now take a piece of thin sheet tin and cut out a piece of the exact size and shape of the base plate; with this difference, that the anterior projection, answering to the handle, should be a trifle larger than in the cup proper; this extra part we turn up into a flange which allows the cup handle to be set down into it, and it serves to keep this plate, which we call the *supplemental cup*, (fig. 3,) in place beneath the true cup when in use. Taking a medium sized hammer with a convex face, and laying this supplemental cup on a counter die, a shallow cavity should be sunk around the centre of the plate *a a*, not exceeding the eighth of an inch in depth. When this supplemental cup or plate is placed beneath the cup proper it will be perceived that a sort of dripping dish has been made; and when the soft plaster is poured into the cup, if any should make its way through the perforations in taking the impressions, it is caught in this receptacle. The supplemental cup thus accomplishes its design.

Fig. 3

When complete, the cup is not so heavy as the ordinary one of the shops. We give two sectional drawings of them as completed and ready for use. Fig. 6 is a section taken from the centre of the anterior projection, and fig. 7, a transverse section, as indicated by the line *c c*, fig. 6. The

Fig. 6

Fig. 7

references are the same in each drawing; *a a* is the base plate; *b b*, the raised portion of the cup; *d d*, the rim or curb; *c c*, the supplemental cup; *i i*, the countersunk apertures in the base plate, underneath which the cavities in the supplemental cup may be observed.

One will necessarily require several of these cups, and to avoid confusion, it would be advisable to number each one and its supplementary cup with corresponding numbers.

In taking an impression, we first take a piece of very fine brass gauze, of about fifty meshes to the inch, cut it in the shape somewhat like the outline of fig. 1, less the handle, about half an inch broader, and a quarter of an inch longer. This gauze is then pressed down into the cup as close to the plate as possible, turning the edge up against the curve. This the pliancy of the gauze will readily permit, after having been properly annealed. Taking out this adjusted gauze, we oil the inner side of the cup, replace the gauze, fill with semi-liquid plaster, and take our impression as usual. On withdrawing the impression, after it has perfectly hardened, any superfluous plaster which may have worked over on the outside of the rim, is trimmed off and removing the supplemental cup with care we find that the plaster has either naturally run through the holes or has been forced through, and meeting with resistance has headed over, firmly fastening the impression, and precluding the possibility of the detachment of the cup when taken from the mouth. Taking the plaster knife, these rivet heads may be cut away, and gently tapping the cup, the impression comes away also.

Another method of taking impressions, or rather of preparing the cup, (communicated to the class of the Baltimore Dental College, December, 1859, by Mr. Bean, of Florida,) is to strike up an impression cup of sheet zinc on an enlarged model of the jaw, and, having given this a coat of melted shellac inside, some clippings of woolen cloth are beaten into it; when the cup is used with plaster these woolen fibres act as minute stays, holding the film of plaster to the cup. This mode, however, is open to the objection that the body of the plaster in the impression, being necessarily very thin, is liable to crack and break away from the unequal expansion of this corrugated plate in the heating required to melt the shellac in loosening the impression therefrom. This, of course, renders it unavailable for our purpose.

Having procured an impression, we now proceed to determine the requirements of the case as to a cavity; if one is needed, what shall be its character and shape; and where shall it be placed? * * * But it would occupy too much space to detail the various requirements which may be met with in the kaleidoscopic presentations of individual cases. We may, however, offer the following general classification:

First.—In mouths in which the alveolar arch is very prominent, both on the palatine and on the buccal exposure, and in which the posterior lobes of the alveolus are very marked, we need only a good fit.

Second.—Where the development is the same as above, with the difference, that the buccinator muscle has its attachment low down on the buccal exposure of the alveolus, preventing the *return* of the plate far into the exterior of the arch—we must have a very perfect adjustment to the palatine surface of the jaw; this may be accomplished by means of two shallow cavities opposite the palatine exposure of the alveolus, where normally the first and second molars were situated; in connection with these we should have an accurate adaptation to the ridge of the jaw throughout its entire arch.

Third.—Where the palatine arches are shallow, with high attachments of the facial muscles, an accurate fit is sufficient, but if as in the

Fourth category, the facial attachments be low, and the whole jaw is decidedly flat, we have to depend entirely upon a large, well-defined, but shallow vacuum chamber in the central horizontal part of the plate covering the roof of the mouth.

Fifth.—The peculiarity we now arrive at may occur in connection with any of the former classes; it consists in a flabby or spongy development of the muscles and mucous membrane covering the posterior part of the palatine arch; the indications are to add, to those previously mentioned, a light bead, either by swaging or soldering a half round wire to the upper posterior edge of the plate, extending from the inner edge of the alveolus on one side, to the corresponding point on the other side. The bead should be raised to suit the vascularity or looseness of the membrane; this condition of the parts may extend forward, when it will be necessary to enlarge the depressions for the reception of the rugæ.

Sixth.—Under this head are included all those cases, in which, unlike the preceding, the entire membrane, covering not only the palatine but also the buccal surfaces, is of a firm, dense, and unyielding nature. In such cases we will find the adherance of the plate greatly increased, if, in addition to the other expedients, we *chase* the entire palatine exposure of the plate with deep lines.

The next step is to obtain the metallic dies; and this may be done as follows: Take a piece of thin Russia sheet iron, of an average width of four inches, and having cut it as in fig. 5, it should be bent to correspond to the outline of the impression, the smaller opening or end of the so formed tube, being about the eighth or quarter of an inch larger all around than the impression; the two ends are then brought together with a tinner's lock-joint, at the apex of the arch, the seam being outside. (Fig. 9.) We now have a curve whose concavity will give us the requisite inclination for drawing the die. Around the smaller end of this curve, about a quarter of an inch from the margin, we prepare a wire net-work, as shown in fig. 10, and, having placed the apparatus with the wire part down, on some smooth, well-oiled surface, the plaster impression may be placed inside, equidistant from every side. The mixture of plaster, asbestos and water, is now poured around the impression so as to imbed all of it except its face. The plaster, it will be perceived, passing through the net-work, secures itself. As this is partially solidified we should next take some of the same mixture and bank it up with the spatula from the impression to the iron. This accomplished, we have a mould as is represented by fig. 12 and fig. 13. After this has been

*Fig.12* *Fig.13*

slowly dried until it has changed to a faint cream color—when the metal is just fluid, a piece of plate, (fig. 14,) so bent as to direct the stream to the posterior part of the mould, is adjusted and the metal may be poured. When the most elevated portions of the impression have been covered to the depth of half an inch, the piece of plate (fig. 14) may be removed, and we again pour until we have a thickness of two inches. Having procured a good cast, by making use of all the incident expedients, when cool it can be removed, and if there is no very decided undercut to the impression, we may cast a second die from the same mould.

*Fig.14*

Now as to the principles involved in the preceding, it may be rmarked, in opposition to the objections urged against the general practice of taking metallic dies direct from the plaster impression, that it is a well known fact that calcined gypsum, having first been minutely pulverized and then mixed with sufficient water to form a creamy liquid, in passing from the liquid to the solid state, increases about $\frac{1}{350}$ of its bulk. This enlargement is retained, even after the latent caloric has been in a measure parted with. This expansion of plaster is supposed to be taken advantage of, by some, to counteract the shrinkage of metals and metallic alloys, in making dies for swaging plates. This is done by first taking the impression, and therefrom a model in this material; two expansions, it is said, are thus obtained against the one contraction in the metallic die. It is urged that in casting the die direct from the impression, the usual expansion is not allowed; this of necessity, gives an incorrect die. Again, it is objected, that, as it is impossible to dry the mould, the impression cracks when the metal is poured, and the die, consequently, becomes more or less rough.

To these objections, we may, in the first place, refer to the wire gauze in the body of the plaster impression; this, remaining encased, with the obvious precautions as to drying, by its minute ramifying wires retains the impression in perfect shape, and precludes the possibility of fissures or cracks in or upon its surface. The wires being slightly corrugated they expand but imperceptibly longitudinally; this slight expansion of the wires upon themselves, however, influences the whole impression in the aggregate expansion of all the wires.

In the second place, the property of zinc to shrink from the centre to the circumference, is an auxiliary to success; for in pouring, with a little dexterity, the cavity formed by the shrinkage, when cooling, is filled with more metal. The necessary manipulations, to accomplish this last end, will readily suggest themselves in the laboratory.

In order to test the excellence of the die obtained by our method, we constructed an apparatus—too complex for description here—by which we were enabled, with micrometric exactness, to ascertain it to be perfect.

The processes for obtaining a die for the lower jaw are quite similar to those for the upper.

## Figures and Tables

**Fig.1. f1:**
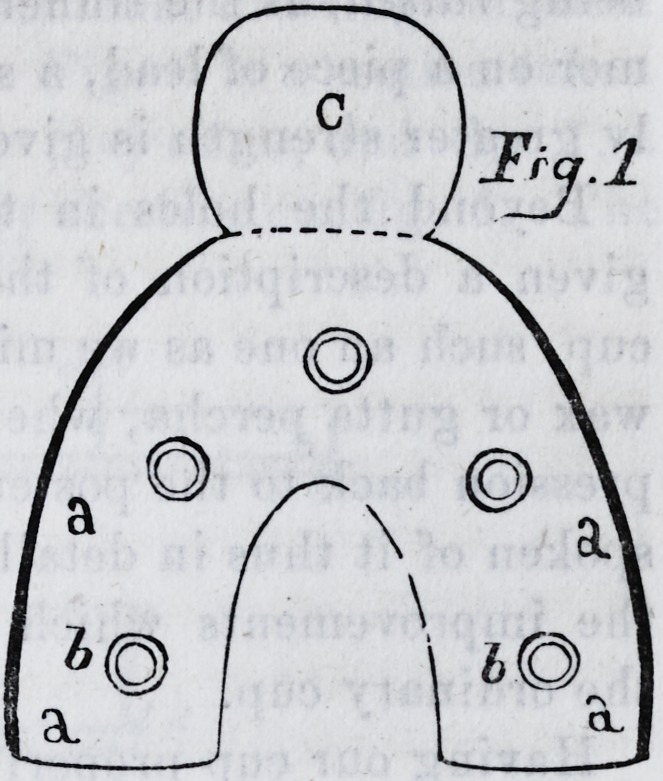


**Fig.4. f2:**
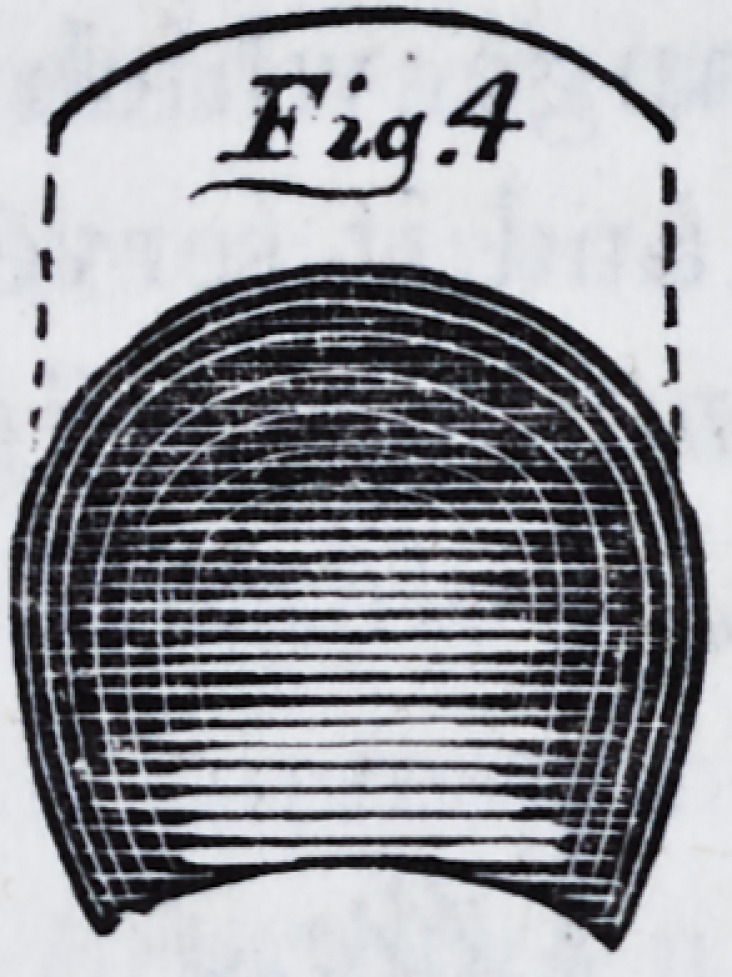


**Fig.5. f3:**
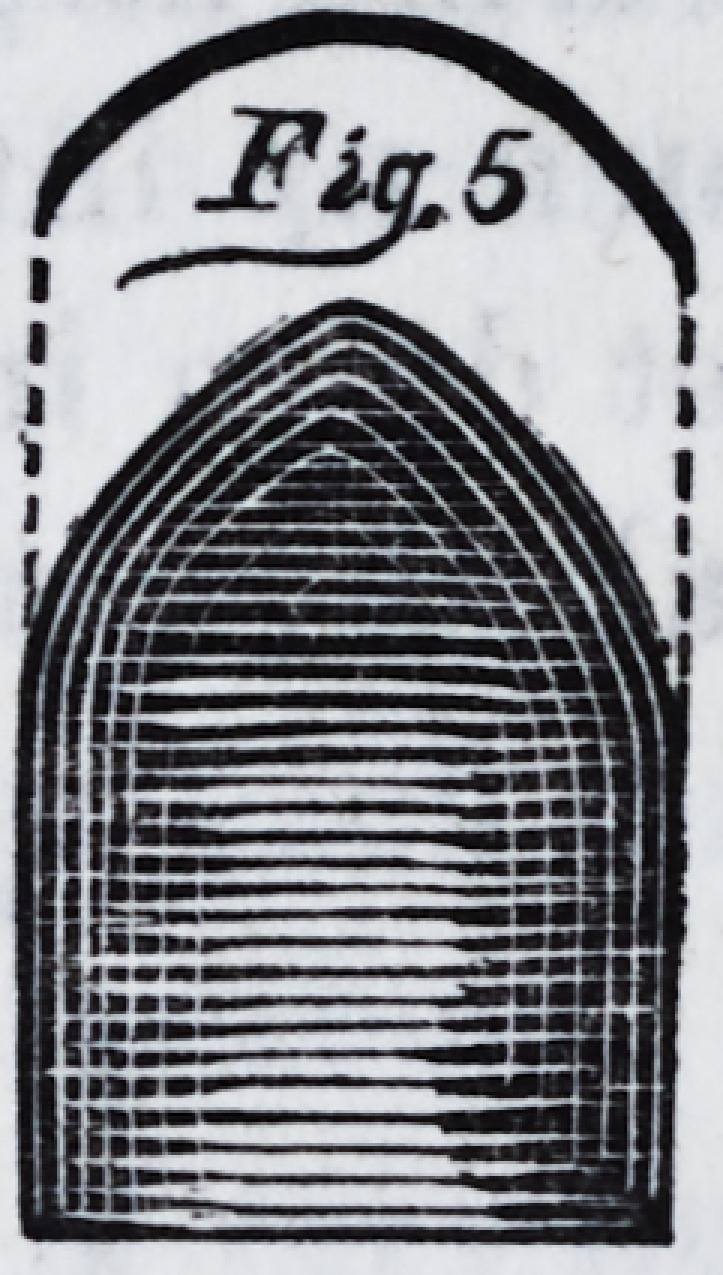


**Fig.2. f4:**
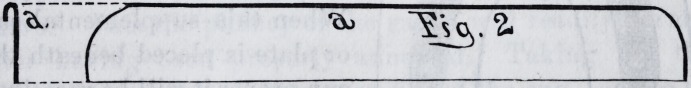


**Fig.3. f5:**
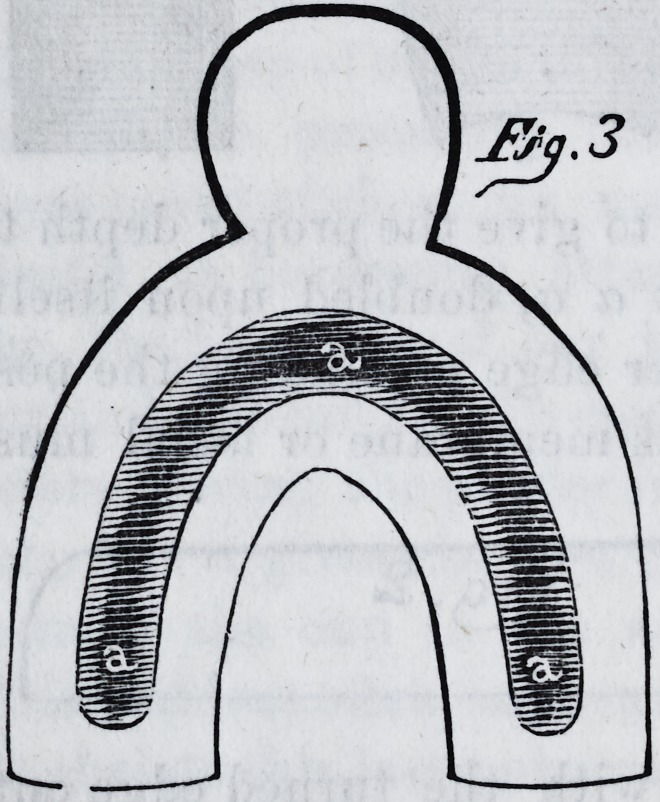


**Fig.6. f6:**
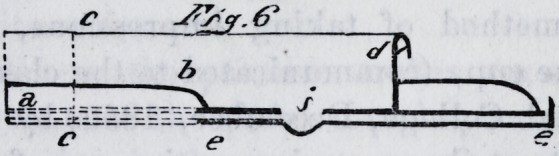


**Fig.7. f7:**
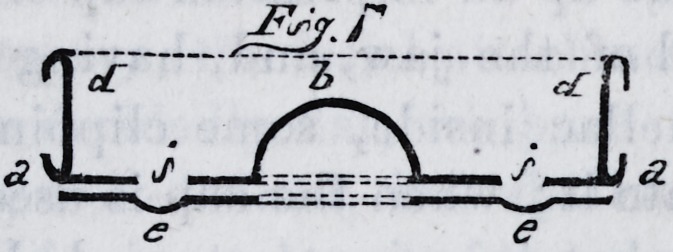


**Figure f8:**
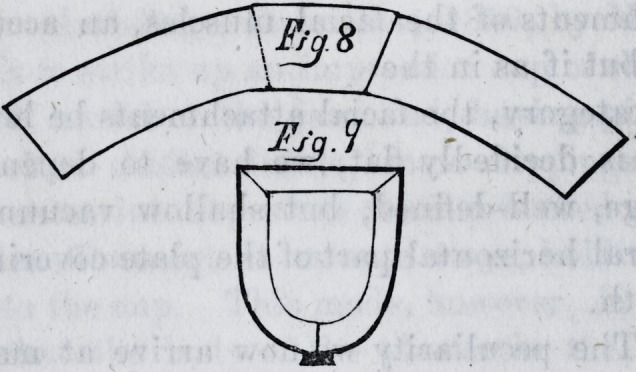


**Fig.10. f9:**
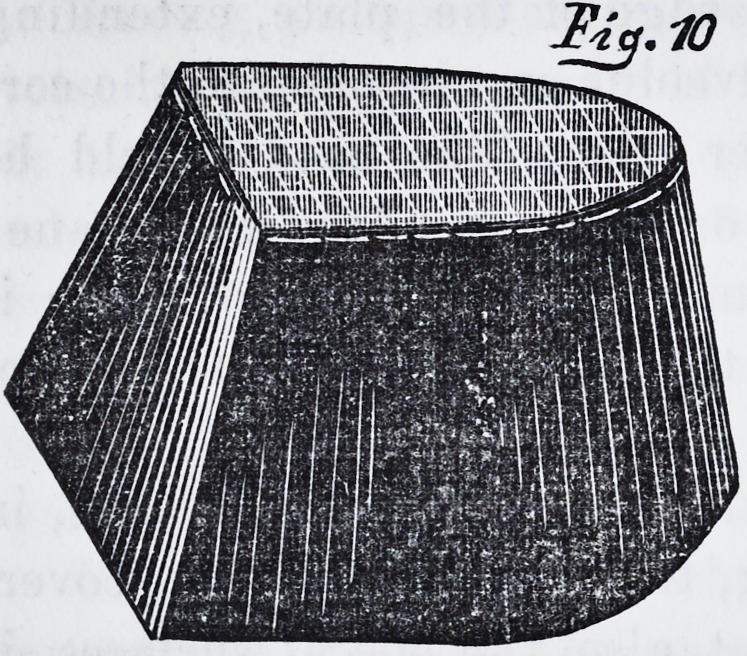


**Fig.11. f10:**
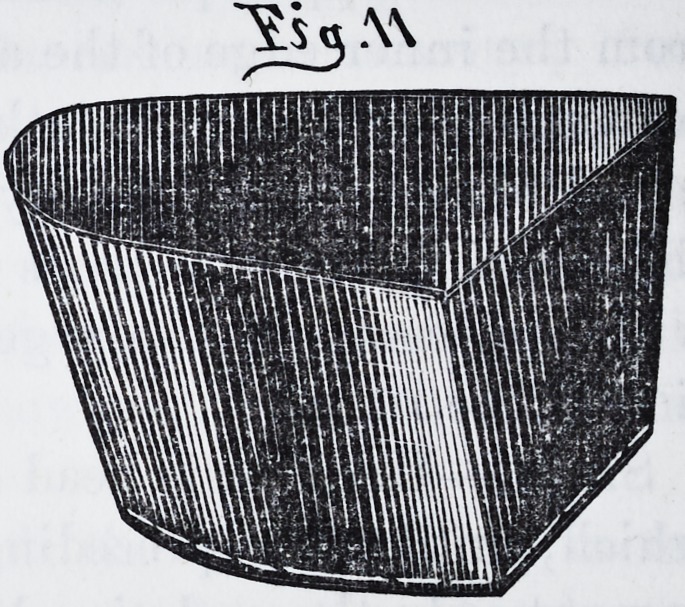


**Fig.12 f11:**
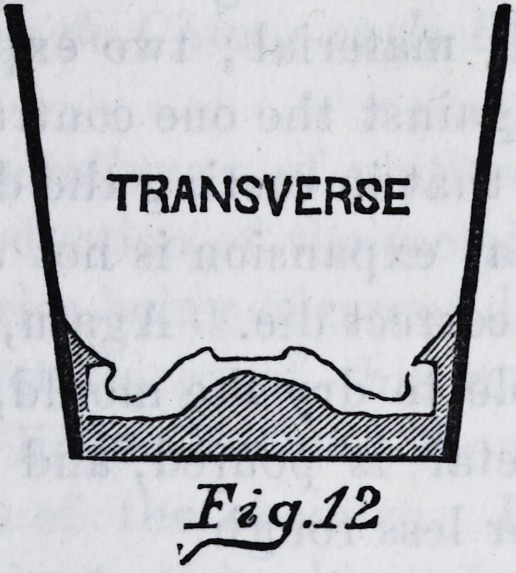


**Fig.13 f12:**
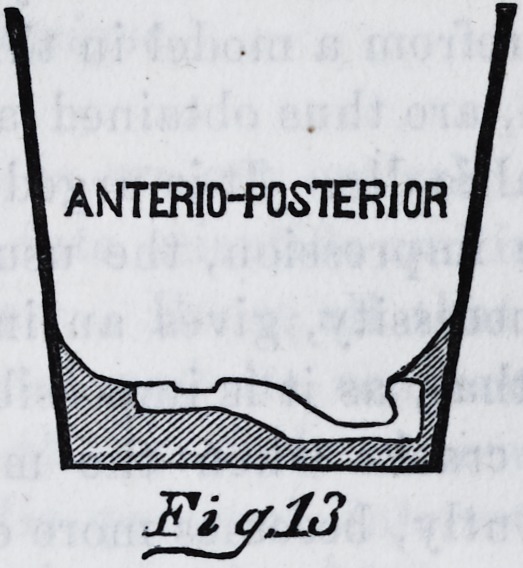


**Fig.14 f13:**